# Protocol for a randomized controlled dismantling study of a brief telephonic psychological intervention applied to non-professional caregivers with symptoms of depression

**DOI:** 10.1186/s12888-015-0682-8

**Published:** 2015-11-23

**Authors:** Fernando L. Vázquez, Ángela Torres, Olga Díaz, Patricia Otero, Vanessa Blanco, Elisabet Hermida

**Affiliations:** Department of Clinical Psychology and Psychobiology, University of Santiago de Compostela, Campus Vida, 15782 Santiago de Compostela, Spain; Department of Psychiatry, Radiology and Public Health, University of Santiago de Compostela, Santiago de Compostela, Spain; Research Group on Mental Health and Psychopathology (GRISAMP), University of Santiago de Compostela, Santiago de Compostela, Spain

**Keywords:** Non-professional caregiver, Indicated prevention, Depression, Telephone intervention, Dismantling strategy, Study protocol

## Abstract

**Background:**

Although depression is a common problem in caregivers and there are effective cognitive-behavioral interventions for its prevention, the ability of caregivers to attend these treatments is often limited by logistics. Furthermore, the efficacy of the components of these interventions is unknown. The objectives of this study are to (a) evaluate the efficacy of a telephone-administered cognitive-behavioral intervention to prevent depression with all its components (cognitive and behavioral) and only with behavioral activation, and to (b) analyze the mediators of the change in depressive symptoms.

**Methods/Design:**

A randomized controlled clinical trial was designed to dismantle the components of a cognitive-behavioral intervention. Caregivers with elevated depressive symptoms will be randomly assigned to a cognitive-behavioral intervention, an intervention with only the behavioral activation component, or a usual care control group. Each condition will consist of approximately 60 participants. The two interventions will consist of five sessions lasting 90 min each, applied to groups of about 5 participants at a time via conference call. Trained interviewers, blind to the experimental conditions, will conduct the assessments at the pre-treatment, post-treatment and 1-, 3-, 6- and 12-month follow-ups.

**Discussion:**

This study will provide evidence of the efficacy of a cognitive-behavioral intervention to prevent depression in caregivers with elevated depressive symptoms administered via conference call, and on the impact of the behavioral activation component on the overall efficacy of the program. If we find favorable results, it would mean that we have developed a program of prevention of depression of higher clinical utility and efficacy than those currently available, which would make it possible for a large number of caregivers to have access to such resources.

**Trial registration:**

ClinicalTrials.gov: NCT02292394. Registered 6 November 2014.

## Background

Across the countries of the Organization for Economic Co-operation and Development (OECD), more than one in ten adults is involved in non-professional, typically unpaid, caregiving, defined as providing help with personal care or basic activities of daily living to people (family or friends) with functional limitations [1]. However, caring for a dependent family member involves emotional consequences for caregivers [2], and can adversely affect their mental health. In fact, it has been found that between 18 % and 48.3 % of caregivers have elevated depressive symptoms [3, 4] and 8.9 % meet the criteria for a major depressive episode [5].

Given their vulnerability, various programs of psychological intervention have been developed for caregivers [6, 7], especially for those with depressive symptoms [8]. However, despite the evidence that having subclinical depressive symptoms is a predictor of developing depression [9], there are only two randomized controlled trials [10, 11] aimed at preventing depression in caregivers with high levels of symptoms that have not yet developed the disorder (i.e., indicated prevention [12]). These interventions achieved a significant reduction in depressive symptoms, but were applied face to face, and face-to-face interventions, pose accessibility barriers to caregivers. Many caregivers cannot participate in face-to-face interventions due to difficulties such as time constraints, lack of financial resources, lack of available and geographically accessible support services, transportation problems, and problems finding a substitute caregiver to the care for the familiar or stigmatization.

To overcome these barriers, communication technologies available to most people in their homes, such as the telephone, can be used to implement the interventions. There is evidence that psychotherapy via telephone for the treatment of depressive symptoms is effective in reducing depressive symptoms compared to control conditions [13]. However, only two studies [14, 15] have evaluated using telephone interventions to reduce depressive symptoms in the caregiver population. They were not indicated prevention interventions for depression, and results revealed that the interventions were partially efficacious in reducing depressive symptoms. Davis and colleagues [14] found no significant differences in depressive symptoms between the face-to-face intervention conducted in home, the phone intervention and the control group. Smith and Toseland [15] only found decreased depressive symptoms in a subsample of caregivers' children. Furthermore, the small sample size, lack of long-term monitoring, the high number of sessions (12 sessions) and interventions that did not follow a theoretical model pose significant limitations that may have contributed to restrict their results.

Moreover, the most effective interventions to reduce depressive symptoms and prevent depression are multicomponent cognitive-behavioral programs [8, 16]. Specifically, in an indicated prevention intervention, Vázquez and colleagues [11] applied cognitive techniques to change thought patterns (e.g., double-standard technique, priming) and behavioral techniques to active behaviors (e.g., pleasant activities planning, reinforcement). They found a significant reduction in the incidence of depression (1.1 % vs. 12.2 %) and depressive symptoms (Cohen's *d* = 1.05) when compared to the control group. Using a dismantling strategy could help identify the contribution of behavioral activation techniques to the therapeutic change. As a result, this can contribute to the improvement of the prevention program and to greater efficiency. The behavioral activation techniques should play an essential role because assuming the caregiver role entails a number of behavioral changes related to leisure and recreation, family life and work that may involve a loss of positive reinforcement and an increase in aversive experiences, factors relevant to the development of depression [17]. In fact, a link between the shortage of leisure activities for caregivers and depressive symptoms has been previously demonstrated [18]. Furthermore, previous research [19–22] comparing antidepressant medication, cognitive techniques aimed at the automatic thoughts and underlying schemes, and behavioral activation techniques have shown that behavioral interventions are sufficient to effectively and efficiently treat depression.

The objectives of the current study are to (a) evaluate the efficacy of a complete cognitive-behavioral intervention (including modification of negative and behavioral activation techniques) and one consisting solely of behavioral activation techniques, both of them administered in a conference call to caregivers with high depressive symptoms; and (b) analyze the mediators of change in depressive symptoms. As the central hypothesis, we expect both interventions to significantly reduce the incidence of depressive episodes and symptoms compared with the control group at post-treatment and at the 1-, 3-, 6- and 12-month follow-up. As secondary hypotheses we expect that (a) the two interventions will differ in the degree of improvement; (b) the change in automatic negative thoughts, self-efficacy will mediate the effect of the complete intervention and the increase in the reinforcement will mediate the effect of the intervention with behavioral component.

## Methods

### Design

The current study will be a randomized controlled clinical trial, wherein a dismantling design will be used. The dismantling design consisted of analyzing the components of a treatment package by removing one component of therapy in one of the comparison groups [23]. Specifically, three groups will be compared: (a) a cognitive-behavioral intervention via conference call (CBC) with cognitive and behavioral techniques; (b) a behavioral activation intervention via conference call (BAC) with only behavioral techniques aimed at activating behavior; and (c) a control group (CG) in which caregivers receive usual care.

**Fig. 1:**
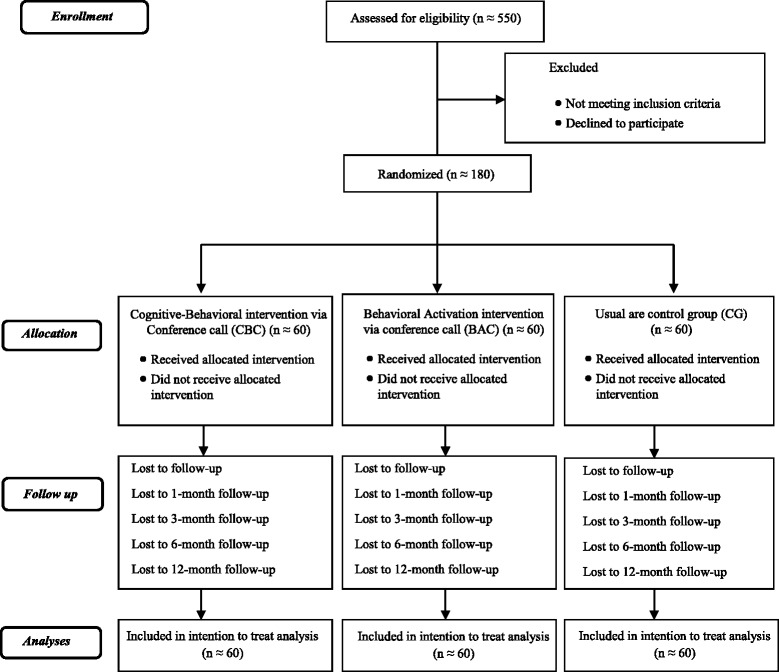
Flow diagram of the progress through the phases of the randomized controlled trial

The phases of the study are shown in Fig. 1. There will be six measurement points in the three groups (i.e., pre-treatment, post-treatment and 1-, 3-, 6- and 12-month follow-ups). The first is baseline (pretreatment). Once the caregivers who meet the eligibility criteria are selected and we administer the interventions, we will conduct a post-treatment evaluation and four subsequent follow-ups at 1, 3, 6, and 12 months. To minimize the loss of subjects, we followed the strategies recommended by Grady and colleagues [24] such as excluding those participants likely to drop out; treat participants with kindness, affection and respect or obtain several contact numbers to be able to track the participant down in the future.

### Sample size

Based on previous studies of indicated prevention of depression in caregivers [10, 11, unpublished paper of Note 1] and studies that have administered therapies for depression by phone (see [13]), we estimate that a sample size of 56 participants per group (168 in total) will be sufficient to detect a difference of 19 % in the rates of incidence of depression episodes between experimental and control conditions, assuming α = .05 and a power (1 - β) of .80. However, assuming an attrition rate of 5-8 %, we estimate that the sample size for each of the groups should be approximately 60 subjects (180 in total).

### Participants, recruitment and eligibility criteria

Participants will be recruited from the Official Register of Caregivers of the Ministry of Labor and Welfare of the Galicia Regional Government in northwestern Spain. A random sample of caregivers stratified by area type [rural (<2000 inhabitants) or urban (≥2000 inhabitants)] and province (Coruña, Lugo, Pontevedra or Orense) will be selected. Previously we have determined that approximately 39 % and 44 % of caregivers have elevated depressive symptoms [10, 11] and 8.9 % have depressive episodes [5]. As a result, to achieve the estimated sample size of approximately 180 participants we will need to randomly select approximately 550 caregivers for a screening prior to the verification of the eligibility criteria.

Caregivers will be contacted by mail and invited to participate in the study. They will be asked to return a stamped postcard if they do not want to be contacted further. Caregivers who do not return that card will be contacted with a brief description of the study. Those who are interested will undergo a screening to assess depressive symptoms and history of depressive episodes. Those who meet the initial selection criteria will be invited to participate in a longer evaluation that will evaluate the inclusion and exclusion criteria.

To be enrolled in the study, participants must meet the following inclusion criteria: (a) be officially recognized by the authorities as caregivers for a relative in a situation of dependency, (b) have a telephone, (c) obtain a pretreatment score ≥ 16 in the Center for Epidemiologic Studies Depression Scale (CES-D [25]), (d) not meet the DSM-IV criteria for a major depressive episode [26], (e) not have a history of major depression, (f) give informed consent. We will exclude those subjects that at the time of the assessment: (a) have been receiving psychological or pharmacological treatment for the last two months, (b) experience other conditions that could act as confounders in the study (e.g., symptoms due to the direct physiological effects of a substance or a medical condition), (c) have medical and mental disorders of such severity that they require immediate intervention (e.g., suicidal ideation) or that would make it impossible to participate in the study (e.g., severe hearing impairment), (d) care for a family member that has a terminal diagnosis, (e) are planning to institutionalize the family member or to move.

### Randomization

Those subjects who meet the eligibility criteria and have signed the informed consent forms will be randomly assigned to the CBC, BAC or CG groups by an external statistician using a table of random numbers. The scrambling sequence will be communicated to researchers through numbered sealed envelopes, one for each participant, with instructions to use them in numerical order.

### Interventions

The manuals for the intervention protocols will be developed during the first months of the project. Psychologists who have been trained by two experienced clinicians, each with over 20 years experience in cognitive-behavioral therapies, will administer the interventions. Therapists will receive about 35 h of training consisting of theoretical and practical seminars and role-playing exercises. They will administer the interventions following the treatment manuals and will be monitored weekly. Each intervention will be administered via conference call and will consist of five 90-min group sessions once a week. All sessions will be recorded and the clinicians involved in the training of therapists will use the recordings to assess adherence to established protocols.

#### Cognitive-Behavioral Intervention via Conference Call (CBC)

CBC is an intervention based on the multifactorial integrative model of depression by Lewinsohn and colleagues [17], which considers depression as the outcome of behavioral, cognitive and emotional changes initiated by the environment and moderated by cognitive factors. The intervention will be adapted from a previous intervention of indicated prevention of depression applied face-to-face in a group format [11]. During the intervention the participants will be trained in various cognitive and behavioral skills such as self-monitoring of mood and activity, relaxation techniques, self-reinforcement, increasing pleasant activities, behavioral contracts, techniques aimed at increasing positive thoughts and decreasing depressogenic ones, training in assertive communication and strategies to increase social contacts.

#### Behavioral Activation Intervention via Conference Call (BAC)

BAC will also be adapted from the program developed by Vázquez and colleagues [11]. However, the intervention will focus on the behavioral activation component of the intervention. During the intervention the participants will be trained in different behavioral strategies such as self-monitoring of mood and activity, self-reinforcement, increasing pleasant activities, behavioral contracts, and training in assertive communication and in strategies to increase social interactions.

#### Usual Care Control Group (CG)

Usual care will constitute the control condition. People assigned to this group will receive no intervention or psychoeducational material. However, we will offer them the possibility of accessing any medical or psychological treatment (public or private) available in their community to treat depressive symptoms.

### Outcomes

Throughout the study, we will collect information on sociodemographic characteristics and situation of care, major depressive episode and other mental disorders, depressive symptoms, the mediating variables (negative thoughts, self-efficacy, reinforcement, social contacts), adherence to treatment, dropouts and satisfaction with the interventions (see Table 1). The self-administered instruments will be mailed to participants to complete them in their homes. The hetero-administered ones will be administered via telephone by trained evaluators who are unfamiliar with the purpose of the study, the interventions that will be administered and the random assignments into each group. Training of the evaluators will consist of 15 h of theoretical and practical workshops and role-playing on the instruments of measurement and evaluation strategies by one of the study investigators who has over 20 years experience in evaluation.

**Table 1:** Overview of measures

Instrument	Intervention phase	Format
Participant characteristics		
Socio-demographic and care situation characteristics	Pre-treatment	Self-administered
Primary outcome		
Major depression: SCID-CV	Pre-treatment, Post-treatment, 1-, 3-, 6- and 12-month follow-ups	Hetero-administered
Secondary outcomes		
Depressive symptoms: CES-D	Pre-treatment, Post-treatment, 1-, 3-, 6- and 12-month follow-ups	Self-administered
Automatic negative thoughts: ATQ-N	Pre-treatment, Post-treatment	Self-administered
Reinforcement: EROS	Pre-treatment, Post-treatment	Self-administered
Register of social networking	Pre-treatment, Post-treatment	Self-administered
Self-efficacy: GSES	Pre-treatment, Post-treatment	Self-administered
Dropout and treatment adherence	During the intervention, Post-treatment, 1-, 3-, 6- and 12-month follow-ups	Hetero-administered
Satisfaction with the service received: CSQ-8	Post-treatment	Self-administered

*Note*: *SCID-VC* Structured Clinical Interview for DSM-IV Axis I Disorders, Clinician Version, *CES-D* Center for Epidemiologic Studies Depression Scale, *ATQ-N* Automatic Thoughts Questionnaire, *EROS* Environmental Reward Observation Scale, *GSES* General Self-Efficacy Scale, *CSQ-8* Client Satisfaction Questionnaire

#### Socio-demographic and care situation characteristics

Through the Characteristics and Status of Caregiver Questionnaire used in previous studies [10, 11] the following information will be collected: sex, age, marital status, social class, level of education, main occupation, relative cared for, gender and age of the person receiving care, duration (in years) of care and daily hours dedicated to care.

#### Primary outcome measure: major depression

Major depression will be diagnosed with the help of the Structured Clinical Interview for DSM-IV Axis I Disorders, Clinician Version (SCID-CV) [27]. This is a semi-structured interview providing DSM-IV diagnostics and consists of six modules (mood episodes, psychotic symptoms, psychotic disorders, mood disorders, substance use disorders, anxiety disorders, and other disorders). In the initial assessment we will use the entire interview, whereas only the major depressive episode module will be used in the subsequent assessments. The SCID-CV has good test-retest reliability and adequate reliability for psychiatric patients (kappa index = 0.61).

#### Secondary outcome measures

##### Depressive symptoms

We will evaluate the severity of the depressive symptoms using the self-reported scale CES-D [25] (Spanish version of Vázquez and colleagues [28]). This scale consists of 20 items in which the person reports how often they experienced each symptom in the last week by a Likert scale with 4 response options ranging from 0 (rarely or none of the time) to 3 (most of the time). The total score ranges from 0 to 60 and higher scores correspond to greater depressive symptomatology. Its internal consistency ranges from .85 to .90, with the Spanish version having a score of .89.

##### Automatic negative thoughts

The occurrence of automatic negative thoughts will be evaluated with the Automatic Thoughts Questionnaire (ATQ-N) [29], which has been translated into Spanish by following the guidelines of Guillemin and colleagues (using the translation / back-translation method) [30]. This questionnaire consists of 30 items in which the participant must indicate the frequency with which they experienced the thoughts during the last week on a Likert scale ranging from 1 (never) to 5 (always). The score ranges between 30 and 150 with a higher score indicating more negative thoughts. The internal consistency for the ATQ-N is .96.

##### Reinforcement

We will use the self-administered Environmental Reward Observation Scale (EROS) [31] (Spanish version of Barraca and Pérez-Álvarez [32]) to assess reinforcement. It has 10 items in which the participant evaluates the degree of positive reinforcement received contingently from the subject’s environment on a Likert scale ranging from 1 (strongly disagree) to 4 (strongly agree). The total score ranges from 10 to 40, with a higher score indicating a higher degree of positive reinforcement. The internal consistency of the Spanish version is .86.

##### Social contacts

The Register of Social Networking (developed and used in the previous study by Vázquez and colleagues [11]) will be used to assess the number of participants' weekly social contacts. In this instrument the participants are asked to report the number of people they have had contact with daily.

##### Self-efficacy

The self-administered General Self-Efficacy Scale (GSES) [33] (Spanish version of Baessler and Schwarzer [34]) will be used to evaluate the self-efficacy of the caregivers. Through its 10 items, each participant assesses the feeling of personal competence to handle difficult situations on a Likert scale ranging from 1 (false) to 4 (true) with higher scores indicating greater self-efficacy. The internal consistency of the Spanish version is .81.

##### Dropout and treatment adherence

We will construct a registry of dropouts from each group over the duration of the study. In addition, treatment adherence will be assessed by recording the number of sessions each caregiver attends and whether they complete homework assignments.

##### Satisfaction with the service received

We will evaluate the participants' self-reported satisfaction with the services received using the Client Satisfaction Questionnaire (CSQ-8) [35] (Castilian version of Vázquez and colleagues [36]). It has 8 items with 4 possible answers and a total score ranging from 8 to 32, with a higher score indicating higher satisfaction. Its internal consistency ranges between .83 and .93 [37].

### Data management

Participant files are to be stored in numerical order and stored in a secure and accessible place and manner. Participant files will be maintained in storage for a period of 5 years after completion of the study. All data will be entered in a database; in which no individuals can be identified. Range checks and consistency checks against data already stored in the database will be made. All forms, audios and hardware related to study data will be kept in locked cabinets. Access to the study data will be restricted. A password system will be utilized to control access. A complete back up of the primary database will be performed twice a month. All reports will be prepared such that no individual subject can be identified.

### Statistical analyses

All analyzes will be conducted in accordance with the principle of intention to treat. All participants will be analyzed in the group to which they were assigned. The most conservative approach for attrition will be adopted by considering dropouts as a treatment failure (i.e., a major depressive episode was triggered) and by replacing missing data with the corresponding baseline scores. Cumulative incidence rates of depression for all three groups will be calculated to examine the efficacy of the interventions on prevention of major depressive episodes. The relative risk (RR) and number needed to treat (NNT) will be calculated according to the formulas proposed by Guyatt et al. [38]. Also, the time it takes for the participants to suffer a major depressive episode will be analyzed using a survival analysis. The effect of the interventions on depressive symptoms will be examined by using mixed factorial analysis of variance (ANOVA) tests with repeated measures. To examine the relationship of the received interventions and depressive symptoms as mediated by change in automatic negative thoughts, self-efficacy, and reinforcement, we will utilize the method recommended by Baron and Kenny [39]. The acceptability and adherence to interventions will be examined by using the Chi-Square test to compare the percentages of dropouts and the Student *t*-test for independent samples will be used to compare session attendance and homework completion. The level of participant satisfaction with the interventions will be analyzed with frequency distribution of each item in the CSQ-8, and we will compare results across both conditions using the Student *t*-test for independent samples. All analyzes will be conducted using SPSS for Windows, version 20.0.

### Monitoring

A Data Monitoring Committee (DMC) will be established, which is independent of the study organizers. Furthermore, the steering committee, led by the principal investigator, will follow the principles of good clinical practice, including the quality control of the clinical protocol and data management and the organization of team meetings. An annual report will be supplied, in strict confidence, to the DMC about the trial development.

A pilot study will be conducted to assess the feasibility of the protocol, interventions and instruments. Any major modification to the protocol which may impact on the conduct of the study, potential benefit of the patient or may affect patient safety, including significant changes of study design, patient population, sample sizes or study procedures will require a formal amendment to the protocol. Such amendment will be approved by the Bioethics Committee prior to the implementation.

In addition, an interim-analysis will be performed after the pilot study and on the primary end point when 50 % of patients have been randomized and have completed the follow-ups. The interim-analysis is performed by an independent statistician. The statistician will report to the independent DMC, who will have unblended access to all data and will discuss the results of the interim-analysis with the steering committee in a joint meeting. The steering committee decides on the continuation of the trial and will report to the Bioethics Committee.

### Ethical issues

All human rights and the dignity of the subjects of the study will be protected according to the Declaration of Helsinki. All procedures in the study have been approved by the Bioethics Committee of the University of Santiago de Compostela (Spain). All participants will be guaranteed confidentiality and will need to give informed consent (first verbally by phone and then in written form using a form that will be mailed to them). Participation in this study will be completely voluntary and participants will not receive any kind of incentive, financial or otherwise.

During the course of the study, if a caregiver develops a major depressive episode, they will be removed from the study and referred to mental health services available in the community for treatment.

## Discussion

In this study we will evaluate the efficacy of a brief cognitive-behavioral intervention of indicated prevention of depression and one consisting solely of behavioral activation techniques, both of them administered in a conference call to caregivers. The prevention programs applied in this study were adapted from a previous study by Vázquez and colleagues [11]. Based on their results, we expect to find a significant reduction in the incidence of depression and depressive symptoms in both intervention groups when compared to a usual care control group.

The use of a conference call to administer psychological interventions as an alternative to traditional face-to-face programs will increase the accessibility to mental health services and increase the tools available to professionals to reach a larger number of people, following the recommendations of National Institute of Mental Health Psychosocial Intervention Development Workgroup [40] and the New Freedom Commission on Mental Health [41]. The advantages of interventions delivered through via phone include anonymity, lower cost and increases in accessibility [42]. Similarly to depression therapies administered over the phone [13], we expect both interventions to significantly reduce depressive symptoms of the caregivers and prevent the emergence of new depressive episodes.

Moreover, the dismantling design of the current study will allow the identification of the contribution of the behavioral activation component to the efficacy of the complete intervention program. This is a significant development because until now no such analysis has been conducted in any study aimed at caregivers. Similar to the results found in clinical interventions for the treatment of depression [20–22], it is expected that the intervention of indicated prevention of depression with techniques designed for behavioral activation will obtain results similar to those of the full cognitive-behavioral intervention. In the literature, behavioral activation has been considered effective in treating depression in adults [19], and it has been included as an evidence-based treatment for depression in guidelines released by the National Institute for Health and Clinical Excellence [43]. If the effect of behavioral activation is powerful enough, it would offer significant benefits to clinical practice, such as achieving simpler effective interventions for caregivers, which may be attractive to clinicians as they are shorter and easier to learn and apply. In addition, behavioral activation can be administered by non-specialized personnel after brief training.

The strengths of this clinical trial include the specification of the level of prevention and the selection of participants accordingly, *a priori* sample size estimation, randomized, controlled design with allocation concealment, implementation of an intervention based on a theoretical model of depression (which was efficacious in previous clinical research) and applied using a manual, evaluation of adherence to protocol, the evaluation of the results by trained professionals who where blind to the study conditions, and 12 months of follow-up. For the evaluation of the outcome measures we will use validated instruments with sound psychometric properties (see [44]). The incidence of depression as primary outcome will be assessed with the SCID-CV, which has been used as the gold standard for clinical diagnosis [45]. Depressive symptoms as secondary outcomes will be evaluated with the CES-D, which is the most widely used tool to assess depressive symptoms in the population of caregivers [46]. In addition, the study will be conducted in the community context where the caregivers live, resulting in a high level of generalizability.

In conclusion, this study can inform the understanding of the efficacy of interventions to prevent depression in caregivers and explore the use of alternative formats to increase accessibility of therapies. It will also determine if a simple intervention, with only behavioral techniques, is effective in preventing major depressive episodes. The results of this study will benefit a large number of caregivers in the present and the future, can be widely applied in health and social services.

### Availability of data and materials

Investigators will communicate trial results via publications. The data supporting these findings will be presented in the main papers or can be obtained from the authors upon request.

## Abbreviations

ATQ-N: Automatic Thoughts Questionnaire
BAC: behavioral activation intervention via conference call
CBC: cognitive-behavioral intervention via conference call
CES-D: Center for Epidemiologic Studies Depression Scale
CG: control group
CSQ-8: Client Satisfaction Questionnaire
EROS: Environmental Reward Observation Scale
GSES: General Self-Efficacy Scale
SCID-VC: Structured Clinical Interview for DSM-IV Axis I Disorders, Clinician Version
